# “He Must Die or Go Mad in This Place”: Prisoners, Insanity, and the Pentonville Model Prison Experiment, 1842–52

**DOI:** 10.1353/bhm.2018.0004

**Published:** 2018

**Authors:** Catherine Cox, Hilary Marland

**Keywords:** Pentonville Prison, separate confinement, insanity, chaplains, doctors, experiment, feigning

## Abstract

The relationship between prisons and mental illness has preoccupied prison administrators, physicians, and reformers from the establishment of the modern prison service in the nineteenth century to the current day. Here we take the case of Pentonville Model Prison, established in 1842 with the aim of reforming convicts through religious exhortation, rigorous discipline and training, and the imposition of separate confinement in its most extreme form. Our article demonstrates how following the introduction of separate confinement, the prison chaplains rather than the medical officers took a lead role in managing the minds of convicts. However, instead of reforming and improving prisoners’ minds, Pentonville became associated with high rates of mental disorder, challenging the institution’s regime and reputation. We explore the role of chaplains, doctors, and other prison officers in debating, disputing, and managing cases of mental breakdown and the dismantling of separate confinement in the face of mounting criticism.

“he is troubled in dreams & tormented . . . heard the death watch here this last week”; “while praying, he saw a face with a bright light about it—he was very much frightened”; “impressed with some strange ideas about a bird”; “the man’s system seems to me to be very low and his mind too much engaged with [Other P-78] one subject”; “exceedingly depressed on his religious state & under temptation to make away with himself”; “he must die or go mad in this place.”^1^

These are a small sample of the disturbing reports of terror and madness experienced by men incarcerated in Pentonville Model Prison after it admitted its first convicts in 1842, as recorded in the journal of the prison chaplain, Rev. Joseph Kingsmill. Kingsmill and his fellow prison officers reported and exchanged opinions on an almost daily basis relating to alarming instances of mental breakdown, delusions, hallucinations, panic, depression, anxiety, and morbid feelings. Prisoners described how they were visited by the spirits of dead relatives, were being poisoned, that the contents of cupboards moved by themselves, or that “things” crawled out of the ventilation system. They wrote strange letters home and became convinced that they were dying, and many attempted suicide or to harm themselves. At night the silence of Pentonville was punctuated by prisoners’ screams. These cases not only were distressing, difficult to manage, and disturbing to the order of the prison, but also threatened to disrupt the “experiment” of separate confinement that had been put in place in Pentonville. This intended not merely to punish but, through imposing rigorous management of prisoners’ movements and activities, solitude and silence combined with industrial training, moral education, and religious teaching and exhortation, to reform, improve, and reeducate the convict population. Though many observers criticized Pentonville’s regime even before it took in its first convicts as being designed to produce mental stress, its supporters lauded the system of separate confinement, which they argued had the potential to produce true and inner reformation and to improve the minds of convicts.

Little historical work has focused on the prison as a site of severe mental disorder, though it is widely acknowledged that many mentally ill people ended up in prison or that their illness was provoked or exacerbated by the prison regime.^2^ Historians and criminologists, meanwhile, have commented on the significance of Pentonville, as emblem and practical, [Other P-79] operational embodiment of the modern penitentiary and the separate system in England.^3^ In Pentonville, the ideal of separation appeared to reach “its fullest expression in the social relations and spatial structures of the model prison,” and to exemplify Michel Foucault’s notion of “disciplinary power,” producing “moral transformation by carefully controlling time, space and bodies”—and the prisoners’ minds.^4^ While existing literature refers to controversies about the degree to which the system of discipline undermined the mental health of Pentonville’s prisoners, an important factor in its eventual dilution after 1847 into a regime considered more bearable for its inmates, the significance of this episode has not been analyzed in detail. Here we closely examine the ways in which mental disorder was dealt with on a daily basis, and understood, explained, and managed by Pentonville’s officers. While keen to mask the scale and depth of mental distress among the convicts, the prison’s minute and visitors’ books, the chaplain’s journal, and the annual reports of Pentonville’s commissioners offer insight into negotiations—and on some occasions disputes—concerning the state of mind of individual prisoners. Kingsmill, chaplain at Pentonville after 1843, and his deputy, John Burt, were important authority figures within the prison and powerful and public defenders of separate confinement.^5^ However, their claims for the benefits of the separate system were undermined by the high incidence of insanity, and Kingsmill, who closely related mental well-being with the capacity of the mind to be reinvigorated as he pursued his initiatives to educate the prisoners, was himself to cast doubt on the benefits of separation. The institutional records reveal many more cases of insanity than appear in Pentonville’s published reports, and confirmed the views of its [Other P-80] critics that, rather than reforming and improving the morals and minds of the convicts, the prison drove them mad. In practice, the high incidence of mental disorder confounded the prison officers’ objectives for order and discipline, producing instead chaos, relaxation of discipline, disputes between the prison officers, and the embarrassment of failure and public condemnation, as well as revealing the capacity of the convicts to subvert the regime by feigning mental breakdown.

In exploring the role of chaplains, doctors, and other prison officers in managing mental disorder and the later dismantling of separate confinement in the face of mounting criticism, this article argues that, during the 1840s, it was prison chaplains, rather than prison doctors, who articulated theories and methods intended to improve the minds of the convicts, based largely on spiritual reform. The separate system was lauded by a number of influential prison chaplains—at Pentonville, Reading, and Preston, for example—not only for its ability to produce deep-seated and sincere reform and redemption, but also for its potential to strengthen mental capacity, to make the prisoners both better and smarter men, equipped to meet the challenges of leaving prison. The subsequent failure of the Pentonville experiment, and its close association with the mission of the chaplains, opened the door wider to prison medical officers who by the 1850s were keen to extend their expertise in the management of prisoners’ health, including their mental well-being.^6^

## The System of Separate Confinement

The Model Prison at Pentonville represented the culmination of many years of thinking about the relationship between punishment and reformation, and experimentation with penitentiary regimes and architecture. The roots of the system are found in the work of early prison reformers, notably John Howard (1726–90) and Elizabeth Fry (1780–1845), horrified at the systematic abuses in gaols and their appalling lack of hygiene, and critical of the ineffective nature of existing forms of prison punishment.^7^ Shaped by the combined influences of evangelicalism and Benthamite utilitarianism, reformers sought more refined punishment and work [Other P-81] regimes, albeit with conflicting agendas. While evangelicals sought to save sinners by urging spiritual and moral reform, utilitarians looked for industrious convicts who could support themselves and prisons through work.^8^ By the early nineteenth century campaigners who favored industrial labor in prisons had all but lost out to spiritual reformers who insisted on complete separation and the centrality of reflection and prayer, criticizing prisons implementing labor regimes for distracting prisoners from the spiritual reflection essential for reform. In 1791 Gloucestershire magistrate, Sir George Onesiphorous Paul, introduced a regime of complete separation in his county gaol, with single cells where prisoners worked and reflected on religious tracts, benefitted from the spiritual guidance provided by chaplains, and endured punitive treadwheel exercise and a low diet. Others, such as George Laval Chesterton, governor of Cold Bath Fields Prison in Middlesex (extended 1794), supported the silent system where separation in cells for long periods during the day allowed for spiritual reflection but was combined with associated labor. Within both systems, the chaplains directed the spiritual reform of prisoners and became influential and powerful figures.^9^

The introduction of the separate system and its Pentonville incarnation was most closely associated with two determined advocates, William Crawford and Rev. William Whitworth Russell, ardent supporters of spiritual and moral reform. Crawford was a founder member and secretary of the Society for the Improvement of Prison Discipline and Reformation of Juvenile Offenders in 1815, and a regular visitor to and critic of London prisons. After being commissioned in 1833 by the home secretary to visit and produce a report on American prisons and penal ideas, he became “entranced” by the system in operation at Eastern State Penitentiary in Philadelphia, established in 1829, which combined separate cellular confinement in a purpose-built institution, with “visits from a battery of reformatory personnel.”^10^ In his view, this was superior to the repressive silent system at Auburn Prison in New York, with its associated dining [Other P-82] and labor, where silence was enforced by flogging. In 1830 Russell was appointed chaplain to Millbank Penitentiary, opened in 1816 as a showcase prison with separate cells and unique in being directly administered by central government. In this position, Russell established great power, with scarcely less authority than Millbank’s governor, as he directed the prisoners’ moral and religious education and undertook individual cell visits. In 1831 and again in 1835 Russell gave evidence to Select Committees on prison reform advocating single cellular confinement, and agreeing with Crawford on the superiority of the separate system as exemplified in Philadelphia. In 1835 Russell and Crawford were appointed prison inspectors for London, and in effect chief inspectors responsible for national reform. Their brief included advising the home secretary on new prison rules and plans, and they were vigorous in devising laws and regulations, openly criticizing magistrates and prison governors who disagreed with their sweeping reforms and promotion of separate confinement.^11^

Russell and Crawford would exert a powerful influence on Pentonville’s vision and governance, and, despite strong criticism of its regime from the outset, remained utterly convinced about the efficacy of separation and in a strong position to enforce their views. Besides Crawford and Russell, Pentonville’s board of eleven commissioners included two physician members, Drs. Benjamin Brodie and Robert Ferguson, and Joshua Jebb, surveyor-general of prisons and Pentonville’s architect.^12^ The commissioners superintended Pentonville, reporting directly to the secretary of state, and appointed its governor, principal medical officers, and chaplains.^13^ From the start, however, there were divisions among them. While Crawford and Russell strongly advocated the separate system, Jebb was more guarded in his commitment, supporting a limited stretch of solitary cellular confinement, keen to harness convict labor and committed to the idea of punishment as a deterrent. Such tensions reflected broader disagreements among prison reformers and administrators on the potential of the separate system. [Other P-83]

Despite these differences, in its earliest years Pentonville was synonymous with the separate system. Intended, as its name implied, to serve as a model for all English prisons, it admitted its first convicts in December 1842. It could accommodate over five hundred prisoners in tiered lines of cells radiating from a central block, with prisoners spending most of their days in isolation in individual cells. Pentonville operated “like a machine” with every minute of the convicts’ day, from the first bell at five thirty until lights out at nine, regimented, directed, and observed in meticulous detail, whether at work, at exercise, or in the chapel.^14^ Prisoners were not to communicate with each other, but worked, ate, and slept in their cells, spending almost twenty-three hours of each day there. They were moved through the prison with their faces covered by hoods and seated in chapel in separate closed stalls, while exercise took place in separate airing yards.^15^

The period of separate confinement at Pentonville was set at eighteen months to allow the full application of probation, instruction, and reflection, and to enable the work of the chaplain to take place once the prisoner “was truly humbled and broken down by solitude.”^16^ As Russell and Crawford proclaimed in 1838,

Upon the offender in his separate cell all the moral machinery of the system is brought to bear with as much force and effect as if the prison contained no other culprit but himself. His submission then must be immediate and complete: he will be calm, for there is nothing to ruffle or discompose him; he will be disposed to self communication, for he has no companion but his own thoughts; he will be led to listen with attention and respect to the instruction, reproof or consolation, of his keepers and instructors.^17^

Even at a time when transportation was in decline, Pentonville was regarded as the “portal to the penal colony” and each prisoner admitted with the knowledge that his period of separate confinement would culminate in transportation, as “he bids adieu to his connexions in England . . . he must look forward to a life of labour in another hemisphere.”^18^ During this period of probation, the prisoner was to be trained in a trade and taught by schoolmasters, to prepare him for his new life overseas, [Other P-84] his future condition in the colony dependent upon his behavior in Pentonville. He entered a “new career,” had the opportunity to learn, earn his own bread, and benefit from the “moral and religious knowledge . . . imparted to him as a guide to his future life.”^19^ The system was also described as testing. For that reason, Pentonville’s prisoners were carefully selected with Russell and Crawford steering this process: prisoners were to be first offenders, in good health, and aged between eighteen and thirty-five, fit for the regime and for reform, suitable subjects for the “experiment” that they would participate in, which aimed not to physically punish, but to correct and retrain the mind before it was fully corrupted.

Yet, even as the first prisoners were brought to Pentonville, separate confinement was attacked in the press and by other prison governors. Its critics, many of whom were supporters of the silent regime, lambasted the system as one unlikely to achieve its objectives, as cruel and too testing for the human spirit. Such concerns had already been raised following Milbank’s experiment with the separate system a few years previously. Millbank, with accommodation for twelve hundred prisoners, the largest prison in Europe and costing a phenomenal £450,000, was criticized for “its slipshod execution of a dubious design.”^20^ While modeled in theory on Bentham’s principles of surveillance, the seven pentagons containing the prisoners’ cells were difficult to navigate, the reduced bread rations provoked riots, and its unhealthy location on marshy land resulted in outbreaks of disease and a high mortality rate.^21^ When Rev. Daniel Nihil replaced Russell as chaplain following Russell’s promotion to prison inspector, he antagonized prison staff, and, as stricter regulations enforcing silence among prisoners were introduced in 1840, cases of insanity began to appear. Peter Laurie, London-based politician, social commentator, and president of the Royal Hospitals of Bethlem and Bridewell, fiercely criticized Millbank, claiming at a meeting of the Middlesex magistrates in 1840, that in 1838 “there had been no fewer than 66 prisoners discharged prior to termination of their respective sentences in consequence of ruined health by solitary confinement in this detestable building.” He went on to describe Millbank as a “secret tribunal . . . even worse than the Bastille in France,” claiming that seven prisoners had been sent from the penitentiary to Bethlem “in a state of mental derangement.”^22^ Coming under sustained attack, the experiment was quickly abandoned [Other P-85] and Millbank converted into a depot for transporting convicts to Australia.^23^

By this time, the terrible effects of the separate system at Philadelphia were receiving widespread attention; its critics described the system of denying human contact as akin to torture.^24^ Charles Dickens, devoting a chapter of his *American Notes* (1842) to an assessment of the separate system in Eastern State Penitentiary, described one prisoner he met there as a “dejected heart-broken wretched creature,” his bed looked “like a grave.”^25^ He condemned the separate system as “cruel and wrong,” “this slow and daily tampering with the mysteries of the brain.”^26^ Chesterton, governor of Cold Bath Fields, decried the confidence placed in “Philadelphian dispensation,” commenting how the zeal of American reformers “had blinded them to the ratio of endurance, which the human mind and the physical frame of man are equal to sustain.” Russell, he added, who had launched a furious attack on Chesterton for his loyalty to the silent system, was “dogmatical and arbitrary to the last degree.”^27^ In an 1841 pamphlet on prison discipline, Augustus Such observed that “placing a prisoner in the [Pentonville] Model Prison for three or four years . . . will tend more to make a man a confirmed idiot, than a good and useful member of society.”^28^ The *Times* published numerous articles condemning Pentonville’s regime and remarking on its impact on the mental state of its inmates. In May 1843, less than six months after the prison opened, an editorial described how the new “prison code” produced mingled feelings of “disgust” and “indignation” with insanity a “probable” or even “inevitable” outcome, as prisoners were moved to the asylum, recovered, and then went back to Pentonville to be “driven mad again.”^29^ The Pentonville Prison Act (1842) had anticipated this, specifying that prisoners who showed signs of mental illness were to be reported to the secretary of state and transferred to an asylum, while the prison’s regulations underlined [Other P-86] the importance of vigilant observation by chaplain and surgeon of the “*state of mind* of every prisoner.”^30^

## Separation, Chaplains, and the Mind

Since the late eighteenth century, prison and other forms of institutional discipline had been envisaged as a medical matter on both sides of the Atlantic, and emphasis placed on moral hygiene and the idea that discipline “habituated the mind to order.”^31^ Ignatieff has suggested that this was exemplified by Philippe Pinel’s (1745–1826) substitution of chains at the Bicêtre Asylum in Paris with “a disciplinary regimen of surveillance, hard labor, and submission to rules” and by Philadelphia physician Benjamin Rush’s (1746–1813) assertion that criminality and insanity were medical pathologies that doctors would soon be able to cure, with the cultivation of the moral faculty becoming the work of the medical profession acting for the state.^32^

During Pentonville’s formative years, however, it was the prison chaplains who asserted themselves in disciplining the mind, claiming that they had the closest oversight and most intimate contact with individual prisoners, and the ability to know and understand their mental state. Their approach resonated with the longer tradition of spiritual reflection exemplified in the regime of moral management at the York Retreat, informed by the emphasis of the Quakers on reflection and the search for inner redemption; they also encouraged self-improvement, though in the context of a punitive and rigid prison environment.^33^ The Pentonville “experiment” took place at a time when the role of doctors in the treatment of mental illness was far from established. The adoption of moral management as the principle of asylum therapy in early nineteenth-century lunatic asylums, and the rebranding of “madhouse doctors” as practitioners of psychological medicine, vested asylum physicians with higher status, though the admission of patients and the management of asylums was still strongly shaped by lay managers.^34^ In contrast, prison doctors, rarely specialists in mental disorder, showed less interest in psychological approaches, overwhelmed as they were with the day-to-day challenges of prison work. [Other P-87]

The chaplains, as spiritual guardians and healers, were key figures within Pentonville’s administrative structures and their duties, conducted with alacrity, heavy. In addition to providing services every weekday morning and evening, they were required, on a daily basis, to visit prisoners in their cells, as well as the prisoners held in punishment cells and in sick wards. They were to keep a journal, a character book, and a general register.^35^ The chaplain was to supervise the assistant chaplain, a schoolmaster, and three assistant schoolmasters.^36^ The full-time medical officer, meanwhile, was assisted in his duties by the resident surgeon and infirmary warder, and was to report on the prisoners’ physical and mental health, and the condition of the prison as it pertained to prisoners’ health and diet.^37^ That the chaplain enjoyed greater seniority was reflected in his salary, of four hundred pounds per annum, less than the governor’s (six hundred) but more than the medical officer’s (three hundred).^38^

Summarizing the anticipated impact of the separate system in 1844, the Pentonville commissioners emphasized the care taken to provide suitable conditions for the prisoners and to plan their training, much of which was directed by the chaplains. The size, arrangements, and ventilation of individual cells were designed to facilitate useful labor and to contribute “to the maintenance of the prisoner’s health and cheerfulness.”^39^ While the prisoners were forbidden—under threat of punishment—to communicate with each other, they were assured that they would have access to the prison’s officers, notably the chaplains, at all times. Crawford and Russell claimed that it was this level of access that differentiated the Pentonville regime from the Philadelphia system, with its disturbing cases of mental illness.^40^ In an effort to distance themselves from the brutality and disorder of older unreformed prisons, the prisoners were described as being in a state of “cheerfulness” as they engaged robustly with the regime, and turned their backs on idleness, delinquency, dissolute practices, and ungodliness.^41^ Early published reports, such as that for 1844, were overwhelmingly positive, proclaiming that since Pentonville’s opening most prisoners had improved “in cheerfulness of spirits and resignation to their [Other P-88] punishment,” showing “their cheerful obedience to the prison rules . . . and their gratitude for the treatment they have received under a discipline which combines instruction and reform.”^42^

Yet 1843—Pentonville’s first full year of operation—had proved enormously challenging in terms of the physical and mental health of the prisoners. In a stark example of the varied ideologies held by the Pentonville commissioners, experiments with the prison diet resulted initially in the rejection of the guidance of the prison’s medical officer, Dr. Owen Rees and of the physician commissioners Brodie and Ferguson, who had urged the adoption of a more generous dietary.^43^ Instead prisoners undergoing separate confinement were placed on the meagre No. 1 diet, the prison’s most restricted diet. This had resulted in weight loss and weakness among many of the convicts, who reported that they felt “faint & sinking” through lack of food; “they wished to have more bread.”^44^ Additionally, a considerable number of cases of depression, insanity, mania, and hallucinations presented themselves, many of a religious tone, threatening to disrupt the prison regime and bringing into question the evangelist approach of the chaplain’s sermons and admonishments.

In autumn 1843 matters came to a head when chaplain Rev. James Ralph’s vigorous style of preaching and cell visitation was questioned for producing “morbid symptoms” in the prisoners.^45^ Convict John Reeves (prisoner 84) had been described by Dr. Rees in December 1842 as “somewhat depressed,” and subsequently was employed about the prison grounds in an attempt to relieve his symptoms. This had been to no avail, and he was moved to the infirmary suffering from maniacal symptoms.^46^ At a special meeting of the commissioners held on April 1, 1843, Reeves was reported to be in a precarious state of health, and Rees advised that he be removed from the prison as soon as possible.^47^ Rees also alluded to the frequent “faintings” occurring in the prison chapel.^48^ Though improvements to the ventilation of the prison appeared to reduce these, alarming [Other P-89] incidences of insanity and hallucinations continued to be reported in the minute books, many of a religious nature. Rees explained how one prisoner would not eat his dinner and “talks much upon religious subjects, & fancies he ought to fast,” another declared that Christ visited him “& gives him sensations” and that the “Devil visits him & converses with him in a flame of fire.” Rees requested that several prisoners should be excused chapel and also recommended removing the Bible and other religious books from convicts’ cells.^49^

In December 1843, the commissioners called a special meeting to investigate these cases. Prior to this, the governor of Pentonville, Robert Hosking, had received a letter from Pentonville commissioner Lord Warncliffe, enclosing correspondence from Rees and commissioner Dr. Brodie, instructing him to call Rees before the board to give evidence concerning the indications of “morbid religious feelings” and their potential consequences for the health of the prisoners. Warncliffe had in the meantime communicated with Rev. Ralph, intimating that he had not exercised sufficient caution given the

peculiar circumstances under which the inmates of Pentonville are placed . . . in respect of his ministrations, and intercourse with the Prisoners. That we must insist upon their having books besides religious books, placed always in their cells, that they may enjoy some relaxation from the constant confinement of their minds . . . and that when the medical officers state to him, that they apprehend ill effects from the state of spirits of any prisoner, he must attend to their suggestions, and alter his mode of communicating with such prisoners on religious subjects . . . that this matter is of vital importance . . . and that Mr Ralph is led only by an exaggerated estimate of his duty as Chaplain, into the errors of which we complain . . . what is absolutely necessary to maintain not only the bodily but the mental health of the prisoners, and that we must have a person in that situation, in whom we can place our confidence, for tempering his zeal with discretion.^50^

Chaplain Ralph was not to be that person. When he appeared before the board, he stated that religious teaching had not been the cause of the prisoners’ mental distress, but that he would resign instantly should the board conclude that his actions had influenced or accelerated the cases. His resignation was accepted.^51^ Kingsmill, then the deputy chaplain, was [Other P-90] appointed in his place, with Rev. John Burt as his assistant. While Ralph’s rapid dispatch might have been seen as an opportunity to curtail the chaplains’ influence, they continued to exert power in observing and reporting on the state of mind of prisoners; they had access through cell visits to individual convicts and were responsible for religious teaching and moral and general instruction. The chaplains’ close contact with the convicts meant that they bore witness to numerous cases of mental distress, though Kingsmill and Burt would come to quite different conclusions about how far this was caused by the separate system.

In the early years of experimentation with separate confinement energetic chaplains, Kingsmill and Burt at Pentonville, as well as John Field at Reading and John Clay at Preston, took as their premise the idea that spiritual reform should lie at the basis of prison discipline.^52^ They worked hard to achieve their goals. In addition to leading religious services and preaching sermons before the departure of convict ships, Kingsmill regularly attended sick prisoners and those confined in punishment cells, and went from cell to cell remonstrating with individual prisoners, often spending ten to twelve hours a day at the prison.^53^ As Forsythe has so aptly stated, with the chaplain as the “central actor,” the search for a change of heart, for reformation of the prisoner, was intended to be “not so much from mere calculative avoidance of crime because of its guaranteed pains, but mainly from the permanent sense of revulsion against sin and crime and a love of Christ.”^54^ The spiritual reformers asserted that they “sought to win the trust of prisoners and to allow prisoners in their isolated cells to share their deepest anxiety and guilt so that not only might the past be purged by confession and admission of truth but comfort be given up upon the sure basis of the particular fear and desperation of the individual.”^55^ Approaches varied; Clay at Preston insisted that reflection would convince the prisoner of his moral failings and obligations to the community, while Burt at Pentonville emphasized how the separate system [Other P-91] broke the will of prisoners, and thus the spiritual messenger would have a particular impact on the emotions of the otherwise isolated and suffering prisoner.^56^ The separate system could also achieve more, potentially strengthening the minds of prisoners, as they reignited religious belief, reformed their habits, and improved their prospects. It was a potential route, in fact, to improved mental well-being.

The chaplains produced prodigious amounts of material on the mind as part of their larger studies of the operation of the separate system.^57^ Those appointed as prison surgeons, meanwhile, were neither experts in the management of mental health, nor free from numerous other responsibilities as they dealt with the physical health of prisoners, the medical care of prison staff, outbreaks of disease, supervision of the infirmary, and overseeing the prison buildings, in particular their ventilation and hygiene.^58^ Prison doctors, however, were forced on a daily basis to deal with cases of mental illness, and often diverged from the chaplain, as will be seen in the examples below, on its diagnosis. They also had to cope with the implications of keeping severely ill prisoners in Pentonville and treating them in the infirmary or managing them in their cells, given the reluctance of its governor and commissioners to admit to incidences of insanity, and to move cases of mental breakdown out of Pentonville.

## “True Cases of Insanity”? The Management of Mental Disturbance

In the first eight years of the prison’s operation, the Pentonville commissioners admitted publicly to only fifteen cases of madness.^59^ However, the medical officer’s reports, minute books, and chaplain’s journal showed the incidence to be much higher. In 1844, turning a blind eye to the hallucinations and delusions that had been prevalent over the course of the year, just three cases of insanity were acknowledged in the published Commissioners’ Report. These included two cases described as religious mania, and all three prisoners were subsequently removed to Bethlem, including John Reeves.^60^ The experiences of the first full year illuminate [Other P-92] several features of the new regime and its dealings with cases of insanity, in addition to the powerful influence of the chaplains. Though played down in the official reports, mental illness was to become a key issue for the prison, absorbing a great deal of time, energy, and resources. The physical health of prisoners, in contrast, tended to be fairly good, once a more “liberal” diet was introduced, outbreaks of disease rare, and deaths few.^61^ However, many instances of mental illness were suppressed or hidden, by the creative labeling of cases that clearly involved mental disturbance. Dr. Baly, medical superintendent to Millbank Prison, would reflect in 1852 that “it has been the custom in some prisons to apply the term ‘insanity’ only to the severer forms of mental disorder, and to place those of a less formidable character in a distinct category with the designation of ‘delusions’”; this was misleading in his view, and delusion commonly signified cases “difficult of cure.”^62^ This certainly seems to have been the policy at Pentonville. During 1844 five cases of hallucination or “illusion,” distinguished from insanity in the Commissioners’ Report, were subject to detailed inquiry, and received medical treatment in Pentonville. Three were described as “of weak mind and unfit for the discipline of the Prison,” “cunning and deceitful characters” to boot, and were later removed to Millbank. The two cases remaining in Pentonville were said to have recovered, and, according to Millbank’s medical officer, those transferred to Millbank showed no symptoms of mental illness after leaving Pentonville and were subsequently transported to Van Diemen’s Land.^63^

The Pentonville authorities insisted that the prison was largely effective in protecting the minds of the prisoners rather than driving them mad and that incidences of mental distress did not result from separate confinement. They pursued rigorous inquiries to root out family histories of mental disease or earlier episodes of mental illness prior to imprisonment, inadvertently demonstrating that their own system of selecting “healthy” prisoners was not operating particularly well. Thus when the three prisoners were removed to Bethlem during the first year of operation, it was reported that they were highly susceptible to mental breakdown. A special meeting lasting two days was convened to enquire into convict John Reeve’s “indisposition” in April 1843, involving Rees, Kingsmill, Governor Hoskins, the governor’s deputy, and commissioner Jebb, the principal schoolmaster, the secretary, and two warders, and notes of inquiry were [Other P-93] sent out to persons who had known Reeves.^64^ Reeves was noted subsequently in the Commissioners’ Report to be “very ignorant” and prior to admission he had engaged in drunken and dissolute practices. It was also noted that he had not even been exposed to the full rigor of separate confinement, as he had been working in association outside of his cell. Convict J.H.S. was “an exceedingly ignorant and superstitious man, and of very weak intellect,” a person of “peculiar manners,” and showed symptoms of hallucination after being confined for just ten weeks. “From inquiries which have been made subsequent to his attacks of mania, both by letter and by personal inquiries in the parish to which he belongs, it was ascertained that the family of J.H.S. have been afflicted with insanity, and that the prisoner himself had at times been considered insane.” The final prisoner to be removed to Bethlem, convict W.C., was well-behaved and industrious, and could read tolerably well, but was also described as “shrewd and cunning, and perhaps irritable” and disliked all but religious studies.^65^ Kingsmill carried out many inquiries into prisoners through correspondence with local clergymen; thus it was revealed that prisoner 66 and most of his family had symptoms of insanity, while two of prisoner 80’s sisters were insane.^66^ The comments of the prisoners themselves on the cause of their mental distress were also noted; the attempted suicide of convict Lewis in 1843 was blamed, seemingly by his own account, not on his incarceration, but on the cruelty of his family, “the remembrance of their injustice made him miserable.”^67^

Many latent cases of mental disease were detected in the schoolroom, when prisoners were assessed as being unable to learn or benefit from spiritual interventions. “Danger” might be apprehended under separate confinement, according to Kingsmill, from conditions of mind that included “sullen obstinacy, no interest is taken in any instruction given here” or want of capacity to learn books or trade.^68^ In September 1846 convict 897 spoke to the schoolmaster in “a very strange manner.” The schoolmaster reported that he had not seen anything approaching hallucination, but that the man was of a “class of prisoners who are incapable from some mental weakness of receiving the advantages of education here offered.”^69^ Opinions concerning particular groups of prisoners were related to their background, ability to learn, and behavior in the prison, [Other P-94] rather than the crimes they had committed, which were mentioned relatively little. In December 1850 Kingsmill was called to visit a Welsh prisoner at the request of resident surgeon Mr. Bradley. While he could not detect either derangement or delusion, he described the prisoner as “an ignorant man of low intellect” and unfit for separate confinement, at least in England. Kingsmill went on to suggest that all Welsh prisoners should be put under probation in Wales and visited by Welsh-speaking ministers.^70^

In 1845 the Commissioners’ Report declared the mental condition of the prisoners—as well as their general health—“highly satisfactory.” By this time, Kingsmill was producing evidence in the form of tables exemplifying how improvements in reading and writing had benefitted the prisoners’ mental well-being. Thus, J.H., whose sister was allegedly weak-minded, and who knew only his alphabet on admission, improved his reading and writing and knew some arithmetic when he left Pentonville, and was “Very cheerful; improved in general knowledge.” T.N., who was referred to as being “an idiot” when imprisoned, left able to read and write well.^71^ Kingsmill also reported that two prisoners who had attempted suicide early on in their confinement both improved in knowledge while in Pentonville; “the former became *very cheerful*, the latter regretted to his friends, on their last visit, that he was leaving the prison before he had learned all he wished.”^72^ Kingsmill anticipated that separate confinement would increase rather than destroy “the better sympathies of human nature,” and concluded that improvement in knowledge, with its constant exercise of the intellectual powers, would lead to improvement “of the mind itself.”^73^ In 1846 further successes in schooling and drawing out the convicts’ “natural resources” were noted; good results were reported with regard to two Irishmen, one of whom was “of low capacity and knew little or nothing but his own native tongue,” while the other, according to his local vicar, was “a man scarcely of sound mind.” Both advanced, according to Kingsmill, in knowledge and capacity.^74^

Kingsmill interpreted such changes as tangible proof of the success of his ministrations and reproduced them in his publications: a letter from Port Philip, Australia, written by a former convict in April 1847 declared thanks for his conversion after being in open rebellion against God “for the interest you took—and in your prayers . . . to mix among the poor prisoners, and to bring home the glad tidings of peace and deliverance [Other P-95] to the unhappy . . . and to assure us God is a very present help in trouble; to proclaim liberty to the captive, and to open the prison-doors of our minds.”^75^ Another case, “A soldier, transported for desertion. Came with a very bad character from his regiment, having been frequently punished for misconduct. After a while here, appeared to be sinking into idiotcy; was under observation on that account; suddenly, seemed to shake it all off, and came out a different person altogether; and was, to the end, a most exemplary prisoner.” Kingsmill described how the idiocy “was counterfeited very ably,” but his appeal to him convinced him of the consequences of this conduct. “He was overcome, and afterwards, when speaking with me on the subject of religion—which he gave every evidence of having truly received—he told me, with tears ‘that talking was the turning-point of my life.’”^76^

While acknowledging that imprisonment in general could have a depressing influence, the commissioners claimed that convicts who had served terms in other prisons remarked that they had “more ease of mind” at Pentonville, while “the more intelligent among them” appreciated the advantages of religious and scholastic instruction and the opportunity to learn a trade. The prisoners generally were “as cheerful and as contented as it can be supposed that any individuals could be under the restraints of imprisonment,” though they missed their families and could be “impatient” about delays in transporting them abroad at the end of their term of separate confinement.^77^ Yet separate confinement was acknowledged as a “severe punishment” and the “severest test,” particularly given its duration of eighteen months, and (contrary to early expectations) it was also being imposed on prisoners who had already undergone several terms of imprisonment.^78^ In 1847 the commissioners, enumerating cases of insanity since the prison opened, claimed that after the first year, when there were three, there were no cases in 1844, and one in each of the following years, both of whom had been removed to Bethlem.^79^ Again this reflects the clear distinction that they were drawing between those who were “actually insane” and sent to Bethlem, sometimes after protracted periods of treatment in the prison infirmary, and those prisoners who exhibited signs of “original weakness of intellect” or who labored under delusions. Three of the prisoners noted to be weak-minded in 1846 were removed to [Other P-96] the invalid hulk. An additional nine were listed as having been treated at Pentonville, where they were given a more stimulating diet and employed in outdoor labor, and were noted to have “recovered.”^80^

The day-to-day reporting of the prison officers told a very different story, as they struggled with high incidences of unusual and sometimes violent behavior, despair, fear, and attempted suicide. In the cells, corridors, and offices of Pentonville notes were exchanged, conversations held about the authenticity and severity of cases, reports drawn up, and recommendations made about the convicts’ mental state on a continuous basis. Extracts from the entry in the medical officer’s journal for December 1846 illuminate the extent to which mental disorder was being observed:

He had received a note from the Governor concerning Convict Schwarnenkruze Reg. No. 998 . . . who entertained a notion he was pardoned . . . he fears however that this Prisoner is suffering from mental symptoms. . . . That Convict Riley [previously noted to suffer mental delusions] continues getting worse. . . . Maddox . . . was nervous and unwell . . . he complained this day (13 Dec.) to the Resid. Surgeon that he heard noises of irons in the flue of the cell. . . . Convict J. Williams . . . has been a very tiresome & suspicious prisoner.^81^

The curt entry in the Commissioners’ Report of 1846 on the fortunes of prisoner J.G. (James Graham, prisoner 635), who was eventually removed to Bethlem, masked the complicated procedures and discussions about his deteriorating condition and the varied opinions of the prison officers over the course of several months.^82^ It was noted in the medical officer’s journal and reported at a meeting of the commissioners held on June 7, 1845, that Graham had been carefully examined, was “very hypochondrical, that he has no hallucinations & that his intellect appears just what it was when first received into the Prison.”^83^ However, in early July Kingsmill reported that he found Graham “terribly excited” when he visited him in the infirmary, especially on the subject of his own death and the presence of his mother who he believed was in the room with him. Kingsmill also asserted, sounding a more positive note as far as he was concerned, “that with these delusions there was great remorse for his sins.” Graham had complained soon after coming to Pentonville that there was something the matter with his head, “feeling something eating away his nose,” and asked the infirmary warder “to remain with him from great fear.” After praying with the prisoner, Kingsmill reported that he was soothed and his [Other P-97] delusions diminished “& his fear in reference to death, & its consequences, were much abated, & more consistent with reason.” A subsequent visit assured Kingsmill that Graham was free from delusion. However, in late July Rees reported that Graham had suffered a severe attack of mania. Rees also testified, again shifting the origins of the disorder from the prison regime to the prisoner, that Graham was naturally weak-minded and was “a very bad man”; his delusions about the insects entering his head, and concerning his mother and father’s spirit “are commonly entertained by highly nervous patients.”^84^ A letter had already been drafted to the secretary of state, requesting Graham’s removal to an asylum, but was held back on the recommendation of commissioner Warncliffe. Dr. Seymour was consulted to provide a second opinion and claimed that Graham would recover in a short time with the medical treatment he was receiving. In August Graham was said to be better, but a few months later his condition again worsened; he refused to eat, and was declared to be monomaniacal by the increasingly desperate medical officer Rees. Finally, in September a letter was sent from Pentonville to the secretary of state, and Graham was removed to Bethlem in October 1845.^85^ This complicated case demonstrates, alongside the close involvement of chaplain Kingsmill, how medical officer Rees saw the need for Graham’s removal as increasingly pressing while the commissioners resisted this. It also shows the complex stories and uncertainty behind the bland entries in the commissioners’ annual reports, and the resistance of the prison authorities to seeking authorization to move mentally ill prisoners.

After his admission to Pentonville in November 1845, convict H. Jones (prisoner 1025) set about destroying his cell furniture and blankets, claiming that “stuff” had been placed in his food. In 1847 Chaplain Kingsmill raised further concerns about Jones, who had written an epitaph for himself in his copy book: “Murdered 22^nd^ of May for the cook.” In June the chaplain showed a further note in Jones’s copy book to Dr. Rees, which gave “notice that he would break more windows, &c. and complains his porter is poisoned.”^86^ Rees reported that he found it difficult to form an opinion on his condition, but as a precaution, after consulting with Dr. Bradley, had Jones’s hands bound with flannel after he had created a disturbance in the refractory ward. Jones was visited in the same month on the advice of the Home Office by two of London’s leading psychiatrists, Dr. Munro and Dr. Conolly. They “could not consider him of unsound mind,” declined to provide a certificate of insanity without further [Other P-98] evidence, and recommended “a continuation of care & watching.” It was also noted that Jones had been under separate confinement in Pentonville for nineteen months, beyond the period allowed for probation. Finally Jones was removed to the York Hulk at Gosport, though Pentonville’s visiting commissioners then reported their anxiety about his “future prospects” and his removal from Pentonville, “as there is no decided opinion given as to his being now perfectly sane.”^87^

In the case of neither Graham nor Jones was shamming raised as a possibility, although Graham was described as a hypochondriac on a number of occasions while Rees expressed “puzzlement” about Jones’s case. Pentonville’s officers were, however, vigilant in watching for cases of shamming and feigned or “insincere” suicide attempts, which occurred on a regular basis, suspecting that prisoners feigned insanity to secure relief from the full rigor of prison discipline.^88^ In 1846 three incidences of shamming, expressed in attempted suicide, were reported along with three cases of simulating madness and imbecility.^89^ The process of agreeing that a prisoner was shamming—as with reaching agreement on the diagnosis of insanity—was beset with confusion and differences of opinion, and in 1847 Kingsmill declared, signaling their lack of expertise, that “it must be exceedingly difficult to medical men to discriminate between those of this class who simulate mental disease, and those who may be in a slight degree affected already, and may be counterfeiting more.”^90^ In October 1847 assistant chaplain Burt communicated with the medical officer and chief warder about the case of Joshua Craig (prisoner 1166) who was showing symptoms of excitement. Two days later Burt expressed a “slight suspicion” that Craig was feigning his symptoms, but after further consideration was “more inclined to think that the symptoms were not assumed.” Shortly after Burt reiterated these concerns, after having his view confirmed by the schoolmaster, Mr. Mitchell. Their opinions were then reported to the medical officer, a visitor, and the chief warder.^91^ Dr. Rees did not share the views of the chaplains, however, suggesting that Craig “puts on symptoms of incoherence and that he does not consider [Other P-99] him the subject of mental disease in any form.” In November Craig told his warder “he fancied he was Lord Nelson,” but Rees remained unconvinced of his insanity. In November Craig was placed in a dark cell, in spite of the chaplain’s continuing concerns, which were rebuffed by Rees and governor Hoskins, who also believed that Craig was feigning. Kingsmill again wrote a note to Rees and once again Rees examined Craig, concluding “invents nonsense, said he was the Saviour, but considers he was not impressed with the idea, as his conduct & manner are not that of an insane person, but impertinent.” Finally in December 1847 Craig was removed to the *Justitia* hulk by order of the secretary of state, the governor and Rees still claiming that Craig was feigning insanity, and Rees certifying that he was “free from mental affection.”^92^

Pentonville’s officers emphasized the ways in which feigning highlighted the prisoner’s “unfitness” for the regime and the discipline of separation, their intrinsic weakness was blamed on bad character or “incorrigibility” and, like Craig, such prisoners were punished by confinement in the dark cell. In June 1845 prisoner 683 was given “3 days dark cell punishment diet, for refusing to work at his trade, & to go to bed at the appointed hour, & also for writing nonsense on his waste paper, his object being to create a belief that he is imbecile.” He was to be joined by prisoner 641, who feigned an attempt to commit suicide “by suspending himself by means of his hammock girth, at a moment when he knew an officer was near his cell.”^93^ Convict 2318, George Williams, became the focus of disagreement between the officers after he was brought from the *Warrior* hulk in August 1849, and Burt concluded that he was maniacal. When Burt visited again a short time after, he was quiet and rational and described the impulse that drove him to such outbreaks as “uncontrollable . . . that he was very nearly breaking out and shouting during divine service this morning and has great difficulty repressing his emotions.” Williams was punished time and again for screaming, shouting, and swearing, riotous conduct, breaking windows, striking one of the warders, and threatening other officers, and was placed in the dark cell on several occasions and also flogged. The chaplains, who found the extra punishments inflicted on Williams and others insupportable given the mental strain already imposed by separate confinement, remained convinced that he was suffering from mania, while the governor and medical officer suggested that he was “absolutely incorrigible.” Flogging, it was noted, produced no good effect, nor had any other punishment, and by September [Other P-100] 1849 it was acknowledged that Williams was being kept in a dark cell to keep him quiet rather than to improve his conduct.^94^

## The System Unravelling

Striking examples of the effect of Pentonville’s discipline on the mind were revealed as convicts left the prison. Shortly after Pentonville opened, the surgeon of the *Wye* hospital ship at Chatham declared that its system of separate confinement had made convicts “more fit for an hospital than for dockyard labour,”^95^ and in July 1845 as the convict ship *Stratheden* sailed from Woolwich toward Van Diemen’s Land an extraordinary incident took place on board. Within forty-eight hours of arrival on the ship, nineteen of Pentonville’s convicts were affected with “Epileptic Fits.” The ship’s surgeon-superintendent, Mr. Baker, reported “most of them had three or four.” Particularly damning was the assertion that convicts removed from other prisons did not suffer these attacks, which, Baker commented, were observed “amongst those who came from Pentonville and who had been from eighteen to twenty months in solitary confinement.”^96^ Mindful of such warnings, the Pentonville commissioners attempted to accustom the convicts to noise and association before they boarded ship, moving them to Millbank and assigning them gardening duties or noisy activities such as chopping wood, but with little success. When Pentonville’s medical officer visited the *Eden* in September 1848 he observed that there had been nineteen cases of convulsive fits out of the 193 prisoners from Pentonville, and “less violent symptoms in great number”; the preparatory association was derided as “utterly valueless as a precaution.”^97^

Yet initial reports concerning prisoners transported from Pentonville, including those of John Hampton, comptroller-general of convicts in Van Diemen’s Land, had commented on their “superior quality” (as a former [Other P-101] ships surgeon Hampton had himself witnessed fits among the Pentonville convicts). The Pentonville commissioners asserted that the prisoners did well once transported, apparently benefitting from their period of probation under the separate system. Hampton described how the convicts were “worthy of the establishment from which they were received” and claimed “that their intellect was in a more vigorous and healthy condition than any prisoners he had previously observed.”^98^ Burt cited more laudatory comments in his account of the operation of the separate system: “*I feel bound to state, in the most emphatic manner, that it* [separate system] *did not produce the slightest mental imbecility in any of the 345 men under my charge*, and that their minds were in a much more healthy state than is usual among ordinary convicts.”^99^ Hampton dismissed the behavior of the convicts when boarding ship as “altogether hysterical” and “propagated by imitation.”^100^ Baker commented of the Pentonville prisoners on the *Stratheden* that all landed in good health and were “particularly quiet and orderly men.”^101^

Whether mental collapse, hysteria, or imitation, the incidences on board ship provoked public commentary, adding to the criticisms to which Pentonville was subjected from the very start for producing insanity, for its inability to deal with it, and for attempting to mask these facts. In 1846 Peter Laurie published an account of the impact of separate confinement on the physical and mental health of prisoners in a range of government prisons and gaols in Britain and America.^102^ As President of Bethlem, Laurie’s particular concern lay with the intake of prisoners from Pentonville into Bethlem, and the failings of its brand of discipline; his goal was to show that the separate system was “highly injurious to the minds of Prisoners” as well as dangerous to their bodily health, demoralizing, and costly. “I assert that this system has consigned a large number of Prisoners as Lunatics to Bethlem Hospital, and has been attended with an extent of mortality and disease not to be found in Prisons conducted on the Silent System.”^103^ Despite the prisoners being handpicked, subject to a rigorous medical examination, and in the prime of life, Laurie concluded that its results had been disastrous—numerous cases of insanity and hallucination, a high death rate, and many cases of sickness. Responding [Other P-102] to Hampton’s praise of Pentonville’s prisoners as healthier than most convicts, Laurie dwelt on his statement that it also resulted in “the loss of gregarious habits.” Laurie interpreted this as “reducing picked, strong, stalwart young men to a state of idiocy.”^104^ A year later in a letter to the *Times*, Laurie reported how he had been compelled as president of Bethlem to hear the warrants of the secretary of state read for admission “of the victims of the separate system sent from the two Government prisons, Millbank and Pentonville.” During the past ten years forty lunatics had been sent from Pentonville, compared with fourteen prisoners in the preceding decade; “are the public expected to believe that this fearful increase is not the direct result of the separate system?”^105^ Laurie was an especially articulate example of a diverse group of commentators who “did not think the experiment of Pentonville worth much as a model prison.”^106^

By the late 1840s Chaplain Kingsmill too was expressing reservations about the impact of separate confinement on prisoners’ minds and indeed its ability to secure long-term reformation. He was shocked at the occurrences of fits among the convicts on board ship, concerned about “the decline of the physical and mental energies” of the prisoners and supported the modification of the separate system.^107^ “Its value in a moral point of view has been greatly over-rated,” he declared in his report to the commissioners for 1849, though he believed it still offered the opportunity for reflection and awaking the conscience of prisoners and was the best deterrent against the repetition of crime.^108^ After the sudden deaths of Russell and Crawford in 1847, the remaining commissioners, several of whom had demurred from Russell and Crawford’s more ideological adherence to the system, started to modify it. The solitary period was reduced initially from eighteen months to fifteen, then in 1848 to twelve and in 1853 to nine months.^109^ In 1850, the Directorate of Convict Prisons, comprising Jebb and two fellow ex-army officers, D. O’Brien and H. P. Voules, was established and took over responsibility for Pentonville’s governance. This greatly enhanced Jebb’s influence.^110^ Now, Pentonville was to admit convicts whom the medical officer deemed capable of undergoing one year’s separate confinement and of laboring on public works afterward [Other P-103] and was also to retain “incorrigible convicts.” On leaving Pentonville, convicts were no longer transported but sent to work at public work schemes at Portland, Woolwich, or Portsmouth.^111^

John Burt, Kingsmill’s deputy, was, however, not to be deterred from his advocacy of separate confinement, and in 1852 published his defense of the system in its purest form. Now that Russell and Crawford were dead, few, according to Burt, knew the details of what had occurred within the walls of Pentonville.^112^ He accused Pentonville’s Committee of Visitors, when they tried to prevent the book’s publication, of “an intention to carry on that whole convict service in secret” and “to conceal facts from the knowledge of the public.”^113^ Once the separate system started to be dismantled, Burt argued, the number of cases of mental breakdown increased. The class of men admitted to Pentonville had deteriorated, and the principle of long and continued separation compromised by reducing it from eighteen to twelve months. During the first five years of operation, after the “special circumstances” of the first difficult year with three cases of mania, in the following four years there were only three cases out of 1,627 prisoners.^114^ However, according to Burt’s figures, once the term of separation was reduced to twelve months, the numbers suffering from mania and delusions increased—to reach a total of eighteen in 1850.^115^

A year prior to the publication of Burt’s book in 1852, the ex-Pentonville commissioners, Drs. Brodie and Ferguson, had produced their report on the system, including its effect on the minds of the prisoners. It had a “powerful,” though possibly not durable, impact they concluded, while for some prisoners there “was a real moral improvement.” But “so powerful an instrument” could “be productive of injurious as well as beneficial results,” and resulted in not only hysteria on boarding the convict ships, but many cases of delusion (they gave figures of fifteen such cases in the prison’s first six years). “There are few minds which would not suffer from the monotony and ennui of this mode of existence.” Their explanation for the increase in maniacal cases after 1847 was the absence of Crawford and Russell, who had “themselves selected, with the greatest care, the convicts who were to be sent to Pentonville, rejecting those who did not seem to be, for any reason, fit subjects for the discipline.” Yet, they concluded, the discipline had further deteriorated once the term of separation was reduced.^116^ They also regretted the loss of transportation as a part [Other P-104] of the reformatory process, lamenting “where the hulks are his ultimate destination, it is useless for a convict to be detained thirty or forty weeks at Pentonville.”^117^ In the same year, Dr. Forbes Winslow delivered a more straightforward message, noting in the *Lancet* that “abundant evidence might be adduced to prove that the present . . . scheme has totally failed as a reformative measure.” Despite setting out to exclude idiots and men known to have been insane, he claimed that fourteen per thousand of Pentonville’s prisoners were suffering from mental disorders, a rate that compared with 2.5 percent for adult working men in England.^118^

By the late 1840s both Rees and Kingsmill were commenting on the excessive “irritability” of the convicts. There were frequent suicide attempts; in 1849 one had succeeded and three others made “resolute attempts to obtain their end.” Though there were fewer cases of mania and mental delusion, what was worrying was that many cases occurred months into the prisoners’ confinement rather than shortly after arrival, making it likely that it was the discipline itself rather than any preexisting condition that was causing distress. The attempted suicides, Rees explained,

though made by men who could not be regarded as insane, were of a nature indicating a recklessness and desperation never before observed in Pentonville Prison. They did not occur among incorrigible men of violent character, but the contrary; and deep despondency appeared to have been the forerunner of the desperation which prompted the act.

With respect to the general mental condition of the prisoners, there is an irritability observable which I never before noticed . . . and which has frequently been a source of anxiety to me.^119^

Diagnoses, in the prison system as much as in midcentury asylums, were vague, though Pentonville adopted its own set of descriptors; “irritability” was used to describe the general prevailing mood among the convicts, and also the absence of self-control and inability to adapt to the discipline of the prison in individual cases, as in convict 1642, reported in 1848 as having attempted suicide.^120^ The prisoner declared “that he must die or go mad in this place—he could never endure so long a confinement—had not communicated with his family in Germany, it would break his mothers heart—that all hope was gone of being in Prussia in time to secure his rights of citizenship & property.” Kingsmill concluded that “I scarcely [Other P-105] think that a man so irritable and with so little self control or principle can bear twelve months more of separate confinement. He shewed little or no compunction for his guilt.”^121^

Increased rates of insanity coincided with a rise in mortality and high numbers of medical discharges, indicative, it was argued in the annual reports, of the deterioration in the prisoners themselves, who were older, unhealthier, and more likely to be repeat offenders and “incorrigibles.” In 1850 twenty-eight prisoners were removed from Pentonville on mental grounds “‘as injuriously affected or likely to be so’ by the discipline.” They were noted to be insane, delusional, depressed, suffering from mania, or weak-minded. Though some of these men were said to have been of “unsound mind before admission,” many others became ill several months into their sentences and no previous indication of mental illness was discovered. Four were removed to Bethlem, and others to the invalid hulks or Portland Prison; on leaving Pentonville, most recovered.^122^ By 1852 only five cases of insanity were listed in the annual report, though a further seven convicts had been removed from Pentonville on “mental grounds” as “unfit for separate confinement.” One prisoner had also been, not surprisingly, rejected by the medical officer as he had twice attempted suicide when previously in Pentonville and had been removed as unfit for separate confinement. Additionally, forty-four prisoners had been suspended from separate confinement due to concerns about their mental health. Bradley concluded that the move to increase outdoor exercise in association had reduced the incidence of mental illness, an initiative inspired by the experiences of Wakefield Prison.^123^ In prisons where separate confinement was less stringently enforced, rates of insanity were noted to be lower and confirmed “that any excess of mental disease at Pentonville was due to a difference in the administration of the system as compared with other prisons; in fact, that there was an absolute relation between the amount of mental disease and the rigour with which the separate system was carried out.”^124^ No longer was Pentonville the model—rather it now fell behind other prisons in its management of mental illness. [Other P-106]

## Conclusion

While the British prison system, compared, for example, with that of the United States, apparently rarely used prison populations in medical experimentation, the early years of the model prison at Pentonville can be seen in effect as a large-scale experiment to judge the impact of separation on the mental state of prisoners. This was described even by Pentonville’s commissioners as rigorous and testing, while for Dickens it was a cruel experiment, taxing the limits of the mind and human spirit.^125^ Nonetheless, Pentonville has been referred to as “an enormous success, exciting emulation throughout the county institutions in England and in . . . Europe.”^126^ Despite extensive criticism of the impact of the separate system on the mind, this form of discipline endured in Britain and provided the model for prison systems in the Western world and numerous colonial contexts into the early decades of the twentieth century, though driven increasingly by ideas of rigorous punishment rather than reform.^127^ Even today, though reports into prison welfare repeatedly highlight its toxic effects on prisoners’ minds, separate confinement continues in use in prisons in Britain and much more extensively the United States. Unsettling parallels can be drawn between the impact of separation in the mid-nineteenth century and the widespread reporting of anxiety, depression, confusion, and self-harm among prisoners enduring solitary confinement today.^128^

When viewed through the prism of the mental disturbances that plagued separate confinement from the start, the Pentonville experiment failed. Whichever figures on the incidences of insanity are taken as being accurate or meaningful—those presented by Pentonville’s supporters or detractors and whether we are taking Pentonville in its purest form with eighteen months of separation or after its dilution—the institution was unable to manage the minds of its convicts. Early claims of the benefits of separation on the morals, industry, learning, and deportment of the prisoners were undermined by the regular occurrence of mental distress in many forms, in spite of attempts to blame mental illness not on the [Other P-107] regime itself but on the unfitness of the convicts to undergo it. Evidence from minute books and journals reveal the prison officers’ daily efforts to cope with the madness of Pentonville. Far from being a place of order, rationality, discipline, and unchallenged state power, the prison was marked on a day-to-day basis by the struggle to manage mania, delusion, depression, and despair. And in the end, the worryingly high incidence of mental disturbance—provoked or made worse by the prison’s discipline—was a key factor in the rejection of the purest form of separation.

Yet at the core of this failed experiment lay interesting debates and commentary about the potential of the mind to improve, through regularity, reeducation, and discipline, including of the self, ideas that were also percolating through the asylum system during the early nineteenth century under the banners of reform and moral management.^129^ During the 1840s, these theories were articulated by prison chaplains who emphasized the importance of spiritual reform for mental well-being. Unlike their counterparts in asylums, prison doctors, with a few exceptions, seemed, if not actually disinterested in proffering ideas on improving the mind, then more concerned with questions of management. It is possible though that the failure of the Pentonville experiment, its association with the mission of the chaplains and their investment in separate confinement, opened the door wider to the management of the mind by medical men by the 1850s, as their position in prisons consolidated.^130^ As psychiatrist and forensic expert Dr. Forbes Winslow argued in 1851, prison doctors had a key role to play—one that they urgently needed to take up—in dealing with mental illness.^131^ In line with the development of distinct professional interests as prison medical officers sought to distinguish themselves from psychiatrists and medico-legal experts while continuing to manage the presence of high numbers of mentally ill people in the prison system, prison doctors would indeed have this form of expertise forced upon them, as they spent increasing amounts of time managing mental disorder. Despite the apparently catastrophic results at Pentonville, the principle of separation continued to be the mainstay of the prison system for the remainder of the nineteenth century. So too was the continuing association of this form of prison discipline with mental breakdown. [Other P-108]

## Footnotes

1 The National Archives (TNA): PCOM 2/353, Pentonville Prison, Middlesex: Chaplain’s Journal, 1846–51, January 9, 1847, 39; July 27, 1847, 68–69; January 6, 1848, 99; January 17, 1848, 101; February 15, 1848, 105. This is the only surviving chaplain’s journal; the journal entries are also embedded in Pentonville’s minute books 1842–52 (PCOM 2/84–89).

2 The impact of the nineteenth-century asylum, meanwhile, has been described in detail: see, e.g., ScullAndrew The Most Solitary of Afflictions: Madness and Society in Britain, 1700–1900 New Haven, Conn. Yale University Press 2005Janet Saunders, “Institutionalised Offenders: A Study of the Victorian Institution and Its Inmates, with Special Reference to Late Nineteenth Century Warwickshire” (Ph.D. diss., University of Warwick, 1983) is unusual in exploring both prison and asylum and movements of inmates between the institutions.

3 See IgnatieffMichaelA Just Measure of Pain: The Penitentiary in the Industrial Revolution, 1750–1850LondonMacmillan1978 ForsytheWilliam JamesThe Reform of Prisoners 1830–1900SydneyCroom Helm1987 McConvilleSeán A History of English Prison Administration 11750–1877 London Routledge & Kegan Paul 1981 SeddonTobyPunishment and Madness: Governing Prisoners with Mental Health ProblemsAbingdonRoutledge-Cavendish20071940

4
FoucaultMichelDiscipline and Punish: The Birth of the PrisonLondonAllen Lane1977 OgbornMiles Discipline, Government and Law: Separate Confinement in the Prisons of England and Wales, 1830–1877 Trans. Inst. Brit. Geog. 20 3 1995 295 311quotations on 295, 296. Ogborn provides a thoughtful analysis of why “separation” continued even after the reformative claims of the “separate system” were undermined. See Ignatieff, *Just Measure of Pain* (n. 3) for his argument that punishment was increasingly applied to the mind in the modern penitentiary.

5
KingsmillJosephChapters on Prisons and Prisoners, and the Prevention of Crime3rd edLondonLongman, Brown, Green, and Longmans1854 BurtJohn TResults of the System of Separate Confinement as Administered at the Pentonville PrisonLondonLongman, Brown, Green, and Longmans1852

6
HardyAnneDevelopment of the Prison Medical Service, 1774–1895The Health of Prisoners: Historical EssaysCreeseRichardBynumWilliam FBearnJosephAmsterdamRodopi199559829061250 SimJoeMedical Power in Prisons: The Prison Medical Service in England 1774–1989Milton KeynesOpen University Press1990

7 Roy Porter, “Howard’s Beginning: Prisons, Disease, Hygiene,” and Anne Summers, “Elizabeth Fry and Mid-Nineteenth Century Reform,” both in Creese, Bynum, and Bearn, *Health of Prisoners* (n. 6), 5–26, 83–101.

8 Martin J. Wiener, “The Health of Prisoners and the Two Faces of Benthamism,” in Creese, Bynum, and Bearn, *Health of Prisoners* (n. 6), 44–58.

9 Forsythe, *Reform of Prisoners* (n. 3), 15–29.

10

ForsytheBill Crawford, William (1788–1847) Dictionary of National Biography (DNB) Oxford Oxford University Press 2004http://www.oxforddnb.com/view/article/6646 (accessed July 30, 2015).For the impact of Eastern State’s design, see EvansRobinThe Fabrication of Virtue: English Prison Architecture, 1750–1840CambridgeCambridge University Press1982318345and for U.S. prisons, see RothmanDavid JPerfecting the Prison: United States, 1789–1865The Oxford History of the Prison: The Practice of Punishment in Western SocietyMorrisNorvalRothmanDavid JNew YorkOxford University Press1998100115

11 Bill Forsythe, “Russell, William Whitworth (1795–1847),” in *DNB* (n. 10), http://www.oxforddnb.com/view/article/73632 (accessed July 30, 2015); “Crawford, William (1788–1847)” (n. 10).

12 Clive Emsley, “Jebb, Sir Joshua (1793–1863),” in *DNB* (n. 10), http://www.oxforddnb.com/view/article/14683 (accessed July 30, 2015).

13 Pentonville Prison Act (5&6 Vict. 1842 c. 29). The commissioners were granted the same powers as visiting justices of other prisons. See also House of Commons Papers, [First] Report of the Commissioners for the Government of Pentonville Prison (RCGPP), Made in Pursuance of the Act 5 & 6 Vict., Sess. 2, C. 29, Sec. 13, 1843 [449], 3–4; MayhewHenryBinnyJohnThe Prisons of London and Scenes of Prison LifeLondonGriffin, Bohn & Co.1862113114

14 Ignatieff, *Just Measure of Pain* (n. 3), 3–9.

15 Second RCGPP, 1844 [536], 18–20, Appendix B, No. 3 “Routine of a Day.”

16

HenriquesURQ The Rise and Decline of the Separate System of Prison Discipline Past and Present 54 1972 61 93quotation on 79.

17 Third Report of the Inspectors of Prisons of Great Britain, Part 1, 1837–38, 28, cited in ForsytheBill Loneliness and Cellular Confinement in English Prisons 1878–1921 Brit. J. Criminol. 44 2004 759 770quotation on 760.

18 [First] RCGPP, 1843, 5, Appendix.

19 Ibid.; Second RCGPP, 1844, 5.

20 Evans, *Fabrication of Virtue* (n. 10), 347.

21 Ignatieff, *Just Measure of Pain* (n. 3), 170–73.

22
Times661840

23 Henriques, “Rise and Decline” (n. 16), 75–76.

24

RubinAshley T A Neo-Institutional Account of Prison Diffusion Law & Soc. Rev. 49 2 2015 365 399 388see also Francis Gray, *Prison Discipline in America* (Boston: Charles C. Little and James Brown, 1847).

25 Cited in WilsonDavid Testing a Civilisation: Charles Dickens on the American Penitentiary System Howard J. Crim. Just. 48 2009 280 296quotations on 290, 292.

26

American Notes Times 10 25 1842See also Francis Gray’s condemnation of the separate system, *Prison Discipline in America* (n. 24), for its production of cases of insanity and of death, and general tendency to enfeeble mind and body.

27

ChestertonGeorge Laval Revelations of Prison Life; with an Inquiry into Prison Discipline and Secondary Punishments2 vols., 3rd ed London Hurst and Blackett 1857 1:183 185

28
SuchAugustusRemarks on Prison Discipline and the Model PrisonLondonShaw and Sons184129

29
Times511843

30 See 5&6 Vict. 1842 c. 29 (n. 13), section xxiii; Times51184311241843

31 Ignatieff, *Just Measure of Pain* (n. 3), 69.

32 Ibid.

33
DigbyAnneMadness, Morality, and Medicine: A Study of the York Retreat, 1796–1914CambridgeCambridge University Press19853356

34 For asylum management in this period, see Scull, *Most Solitary of Afflictions* (n. 2), 216–31.

35 Third RCGPP, 1845 [613], 5.

36 Second RCGPP, 1844, 11.

37 Third RCGPP, 1845, 17–23.

38 Ibid., 23.

39 Ibid., 5.

40
Times511843

41 As with the term “sympathy” outlined by McGowen, which “promised to transform social distance into union, social difference into identity”: McGowenRandall A Powerful Sympathy: Terror, the Prison and Humanitarian Reform in Early Nineteenth-Century Britain J. Brit. Stud. 25 1986 312 334quotation on 314.

42 Second RCGPP, 1844, 10–11, 5; Randall McGowen, “The Well-Ordered Prison: England, 1780–1865,” in Morris and Rothman, *Oxford History of the Prison* (n. 10), 71–99.

43 Sir Benjamin Brodie had a prestigious surgical practice and was surgeon to the royal family; Robert Ferguson was physician-accoucheur to the queen. Both had appointments in major London hospitals: London Medical Directory1846

44 Second RCGPP, 1844, 10–11; TNA PCOM 2/84, Minute Book 1842–44, December 17, 1842, 98.

45 McConville, *History of English Prison Administration* (n. 3), 207; TNA PCOM 2/84, Minute Book 1842–44, December 16, 1843, 239.

46 TNA PCOM 2/84, Minute Book 1842–44, December 17, 1842, 98.

47 Ibid., April 1, 1843, 128.

48 Ibid.

49 Ibid., November 25, 1843, 221; December 9, 1843, 231–32.

50 London School of Economics, Jebb Papers: Jebb/3/1 No. 36, Letter from Wharncliff, September 22, 1843, marked “Private and Confidential.”

51 TNA PCOM 2/84, Minute Book 1842–44, Special Meeting of the Board, December 16, 1843, 239.

52 Forsythe, *Reform of Prisoners* (n. 3), 44–71; ClayWLRev.The Prison Chaplain: A Memoir of the Rev. John Clay by His SonLondonMacmillan1861 FieldJohnPrison Discipline, and the Advantages of the Separate System of ImprisonmentLondonLongman1846For Preston Gaol and the influence of chaplain John Clay, see DeLacyMargaretPrison Reform in Lancashire, 1700–1850: A Study in Local AdministrationStanford, Calif.Stanford University Press1986205224

53 Between 1845 and 1855 he carried out over a hundred thousand cell visits. TNA PCOM 2/353, Chaplain’s Journal, 1846–51; Bill Forsythe, “Kingsmill, Joseph (1805/6–1865),” in *DNB* (n. 10), http://www.oxforddnb.com/view/article/56015 (accessed July 30, 2015).

54 Forsythe, *Reform of Prisoners* (n. 3), 45, 48.

55 Ibid., 46.

56 Burt, *Results of the System* (n. 5), 257, 260. See also Henriques, “Rise and Decline” (n. 16), 80, for differences in tone among the chaplains.

57 Kingsmill, *Chapters on Prisons and Prisoners*, 3rd ed. (n. 5); Burt, *Results of the System* (n. 5); Field, *Prison Discipline* (n. 52).

58 Dr. Owen Rees was assistant physician at Guy’s Hospital, an expert on urinary diseases; resident surgeon Charles Bradley was qualified as a general practitioner: London Medical Directory 1846See Hardy, “Development of the Prison Medical Service” (n. 6).

59 Ignatieff, *Just Measure of Pain* (n. 3), 199.

60 Second RCGPP, 1844, 9, 51, 52.

61 Vivienne Hannon, “A Study of the Health of Convicts at Pentonville Prison, 1842–1859” (Intercalated M.Sc. diss., University College London, 2006).

62 House of Commons Papers, Report on the Discipline and Management of Convict Prisons (RDMCP), 1852 (1852–53) [1659], 94–95.

63 Australia’s chief penal colony, later Tasmania. Second RCGPP, 1844, 10, 51.

64 TNA PCOM 2/84, Minute Book 1842–44, Special Meeting, April 10–11, 1843, 103.

65 Second RCGPP, 1844, 9–10.

66 TNA PCOM 2/84, Minute Book 1842–44, July 22, 1843, 199.

67 Ibid., 149.

68 Fifth RCGPP, 1847 [818] [1192], 41.

69 TNA PCOM 2/353, Chaplain’s Journal, 1846–51, September 9, 1846, 15.

70 Ibid., December 8, 1850, 230.

71 Third RCGPP, 1845, 12.

72 Ibid., 13.

73 Ibid., 13, 11.

74 Fourth RCGPP, 1846 [751], 27, 30.

75
KingsmillJosephChapters on Prisons and Prisoners2nd edLondonLongman, Brown, Green & Longmans18528182

76 Kingsmill, *Chapters on Prisons and Prisoners*, 3rd ed. (n. 5), 231–32.

77 Fifth RCGPP, 1847, 11.

78 Fourth RCGPP, 1846, 28.

79 Fifth RCGPP, 1847, 11.

80 Ibid., 12.

81 TNA PCOM 2/86, Minute Book 1846–47, December 19, 1846, 126.

82 Fourth RCGPP, 1846, 9.

83 TNA PCOM 2/85, Minute Book 1845–46, June 7, 1845, 5.

84 Ibid., July 5, 1845, 34.

85 Ibid., Minutes June to October 1845, 22–88.

86 TNA PCOM 2/353, Chaplain’s Journal, 1846–51, May 22 and June 6, 1847, 59, 61.

87 TNA PCOM 2/86, Minute Book 1846–47, June 19, 1847, 304, 309; TNA PCOM 2/87, Minute Book 1847–48, July 17, 1847, 10, 12, 8; TNA PCOM, Sixth Report, 49.

88 While the number of “successful” suicides recorded in the annual reports of the commissioners was relatively low (see O’DonnellIan Prisoners, Solitude and Time[ New York Oxford University Press 2014] 25 26the sources consulted here indicate that suicide attempts—some more determined than others—were commonplace.

89 Fourth RCGPP, 1846, 25.

90 Fifth RCGPP, 1847, 41.

91 TNA PCOM 2/353, Chaplain’s Journal, 1846–51, October 9, 11, and 15, 1847, 83, 85.

92 TNA PCOM 2/87, Minute Book 1847–48, October 23, 1847, 73, 74, November 6, 1847, 83, November 22, 1847, 92, 93, December 18, 1847, 111, 113.

93 TNA PCOM 2/84, Minute Book 1845–46, June 7, 1845, 3–4.

94 TNA PCOM 2/353, Chaplain’s Journal, 1846–51, August 26 and 30, September 6, 1849, 163–65, TNA PCOM 2/93, Visitor’s Order Book, 1849–50, August 4 and 27, September 7, and November 5, 1849, 36, 37, 40 [np].

95 House of Commons Papers, Medical Report of the Fortitude Convict Hulk, *Convicts, Two Reports of John Henry Capper* (1843), [113], xlii, 8, cited in FoxhallKatherineHealth, Medicine, and the Sea: Australian Voyages c.1815–1860ManchesterManchester University Press201235

96 TNA Admiralty Papers (ADM) 101 69/6, Medical Journal of the *Stratheden*, convict ship from July 22, 1845, to January 7, 1846, by Henry Baker, surgeon and superintendent, 1845–46, 2r. See also TNA ADM 101/49/10, Medical Journal of the *Marion*, convict ship, for September 1, 1847, to February 5, 1848, by John Anderson, surgeon and superintendent, 1847–48, 20v.

97 TNA PCOM 2/88, Minute Book 1848, October 14, 1848, 39.

98 TNA ADM 101/67/10, Journal of surgeon John S. Hampton on convict ship *Sir George Seymour* (1844–45), General Remarks; Fourth RCGPP, 1846, 10.

99 Burt, *Results of the System* (n. 5), 109.

100 Fourth RCGPP, 1846, 10.

101 Ibid., 5.

102
LauriePeter“Killing No Murder”; or, The Effects of Separate Confinement on the Bodily and Mental Condition of Prisoners in the Government Prisons and Other Gaols in Great Britain and AmericaLondonJohn Murray1846

103 Ibid., 2.

104 Ibid., 11.

105
Times1111847

106
Times5291847

107 RDMCP, 1852, “Extracts from the Report of the Chaplain of Pentonville Prison for 1852,” 93–94.

108 Eighth RCGPP, 1850 [1192], 16.

109 Senate House Library, B. C. Brodie and R. Ferguson, “Pentonville Prison” (unpublished MS; London: Schulze, 1851), 18; Henriques, “Rise and Decline” (n. 16), 86.

110 McConville, *History of Prison Administration* (n. 3), 215–16.

111 Eighth RCGPP, 1850, 3–4.

112 Burt, *Results of the System* (n. 5), preface.

113 TNA PCOM 2/89, Minute Book 1849–52, November 24, 1852, np.

114 Burt, *Results of the System* (n. 5), 96–97.

115 Ibid., 129.

116 Brodie and Ferguson, “Pentonville Prison” (n. 109), 5, 6, 9, 10.

117 Ibid., 13.

118

WinslowForbes Medical Society of London. Prison Discipline Lancet 3 29 1851 357 360quotation on 359.

119 Eighth RCGPP, 1850, 23.

120 For asylum taxonomies, see RayLaurence JModels of Madness in Victorian Asylum PracticeEur. J. Sociol.222198122926410.1017/s000397560000371411630885

121 TNA PCOM 2/353, Chaplain’s Journal, 1846–51, January 17, 1848, 101.

122 House of Commons Papers, Reports of the Director of Convict Prisoners, for Year 1850 (1851) [1409], 35, 40–41, 43–45.

123 RDMCP, 1852, 39, 33, 37.

124 Ibid., 13.

125
Times511841On experimentation in U.S. prisons, see, e.g., HornblumAllen MAcres of Skin: Human Experiments at Holmesburg Prison: A Story of Abuse and Exploitation in the Name of Medical ScienceNew YorkRoutledge1998

126 Ignatieff, *Just Measure of Pain* (n. 3), 197; O’Donnell, *Prisoners, Solitude and Time* (n. 88), 23.

127 Ogborn, “Discipline, Government and Law” (n. 4); Forsythe, “Loneliness and Cellular Confinement” (n. 17).

128
ShalevSharonEdgarKimmettDeep Custody: Segregation Units and Close Supervision Centres in England and WalesLondonConquest Litho2015

129 Ray, “Models of Madness” (n. 120); Scull, *Most Solitary of All Afflictions* (n. 2), chaps. 2–4 discuss reform and moral treatment in the early nineteenth century.

130 Hardy, “Development of the Prison Medical Service” (n. 6); Sim, *Medical Power in Prisons* (n. 6).

131 Winslow, “Medical Society of London” (n. 118).

